# Understanding Long-Term Changes in Species Abundance Using a Niche-Based Approach

**DOI:** 10.1371/journal.pone.0079186

**Published:** 2013-11-12

**Authors:** Pierre Helaouët, Grégory Beaugrand, Martin Edwards

**Affiliations:** 1 Sir Alister Hardy Foundation for Ocean Science, Plymouth, England; 2 Centre National de la Recherche Scientifique, Laboratoire d’Océanologie et de Géosciences’ UMR LOG CNRS 8187, Station Marine, Université des Sciences et Technologies de Lille – Lille Wimereux, France; 3 Sir Alister Hardy Foundation for Ocean Science, Plymouth, England; University of Waikato (National Institute of Water and Atmospheric Research), New Zealand

## Abstract

One of the major challenges to understanding population changes in ecology for assessment purposes is the difficulty in evaluating the suitability of an area for a given species. Here we used a new simple approach able to faithfully predict through time the abundance of two key zooplanktonic species by focusing on the relationship between the species’ environmental preferences and their observed abundances. The approach is applied to the marine copepods *Calanus finmarchicus* and *C. helgolandicus* as a case study characterising the multidecadal dynamics of the North Sea ecosystem. We removed all North Sea data from the Continuous Plankton Recorder (CPR) dataset and described for both species a simplified ecological niche using Sea Surface Temperature (SST) and CPR Phytoplankton Colour Index (PCI). We then modelled the dynamics of each species by associating the North Sea’s environmental parameters to the species’ ecological niches, thus creating a method to assess the suitability of this area. By using both *C. finmarchicus* and *C. helgolandicus* as indicators, the procedure reproduces the documented switches from cold to warm temperate states observed in the North Sea.

## Introduction

Covariations between climate change and the alteration in the abundance, spatial range and phenology of species have been widely reported [1], [2], [3], [4], [5]. The mechanisms involved, however are not at present fully understood, because both climatic variability and biological dynamics operate at different scales making modelling difficult [6]. Concurrently with the increase in model complexity, new integrative approaches are being defined using the concepts of ecological niche [7]. Considering their own strengths and weaknesses, those approaches are complementary to more traditional mechanistic models [8].

The North Sea, constituting a transitional region between the Atlantic Polar and the Atlantic Westerly Winds biomes [9], is an important productive region providing 5% of the global fish harvest [10]. This region has been monitored by the Continuous Plankton Recorder (CPR) survey for more than 50 years, providing information on geographical distribution, seasonal cycles and year-to-year changes in the abundance of marine plankton. Among species identified by the CPR survey, *Calanus finmarchicus* is one of the most dominant species (i.e. in terms of biomass) of the North Sea mesozooplanktonic assemblage [11] and is indicative of the Atlantic Arctic biome [12]. Its congeneric species *C. helgolandicus* is more indicative of the Atlantic Westerly Winds biomes and the joint information on both species have traditionally been used to describe the state of the North Sea [13]; an increase in the relative proportion of *C. helgolandicus* suggests an alteration of North Sea ecosystems towards a warmer dynamic regime.

Here, we investigate the long-term changes in both *C. finmarchicus* and *C. helgolandicus* by using an approach based on the concept of the ecological niche *sensu* Hutchinson [14]. We first characterise the ecological niche of both species using two environmental factors known to have a cardinal influence on their spatial distribution in the North Atlantic Ocean: Sea Surface Temperature (SST) and the Phytoplankton Colour Index (PCI) [15]. A procedure is proposed to superimpose the monthly trajectories of the North Sea environmental conditions on the niche space of both *Calanus*, which enable us to calculate their expected abundance. Observed and expected patterns in the changes of the dominance of both *Calanus* species in the North Sea were highly significantly correlated, suggesting that the parameters integrated in our procedure explained a large part of the decadal changes in the abundance of the two species. We then study the relationships between both the observed and expected abundance of the two calanoids and reveal that the state of the North Sea has moved from a cold- to warm-dynamic regime at the end of the 1980s.

## Materials and Methods

### Software

All analyses have been programmed using MATLAB® (R2012a 7.14.0.739).

### Studied Area

**Figure 1:**
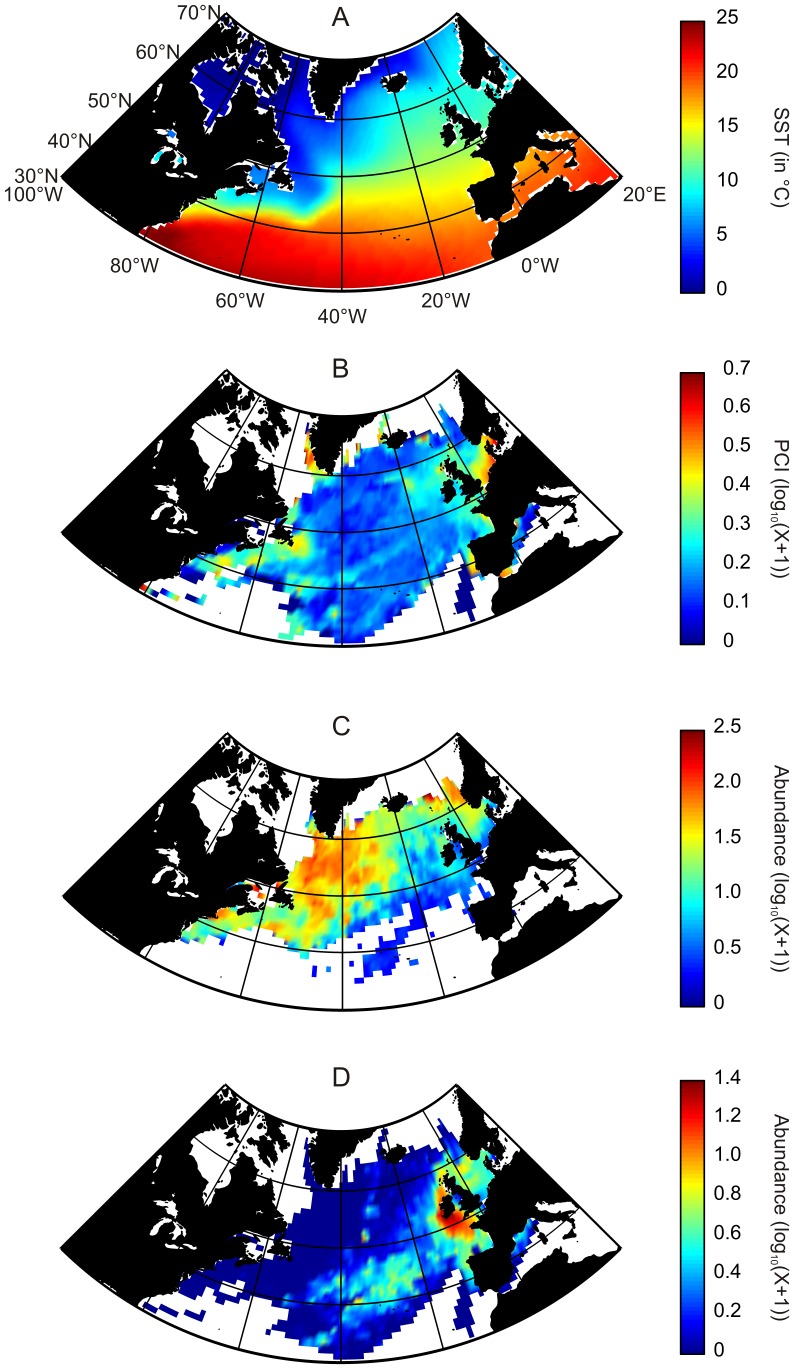
Averaged spatial distribution, over the period 1958–2008, of biological and physical data in the North Atlantic Ocean. (A) Sea Surface Temperature. (B) Phytoplankton Colour Index. (C) *Calanus finmarchicus*. (D) *Calanus helgolandicus*. No interpolation made.

In the North Atlantic, we investigated an area delimited spatially by the longitudes 99.5°W and 19.5°E and latitudes 29.5°N and 69.5°N (Figure 1). In the North Sea, the area was delimited between longitudes 4.5°W and 10.5°E and latitudes 50.5°N and 60.5°N. The time period ranged from 1958 to 2008.

### Data

Previous studies carried on *Calanus finmarchicus* and *C. helgolandicus* have identified two main parameters explaining their distribution in space in time. Sea Surface Temperature (SST) appears to be the main driver while an index of food concentration (e.g. the phytoplankton colour index or the quantity of chlorophyll a converted or not into food concentration) also has a significant structuring power [16], [17], especially at a seasonal scale [15]. Helaouët *et al.* [15] performed a Principal Component Analysis on 11 environmental parameters over the whole North Atlantic Ocean to evaluate the main drivers of this system. The results showed that SST was the main contributors to the first principal component representing 49% of the total variance. Chlorophyll a, used as a proxy for food instead of PCI, has a significant relative contribution to the second principal component representing 20.8% of the total variance.

#### Sea Surface Temperature (SST)

Temperature has a well-documented effect on living organisms by having a central role in metabolic rates [18], [19]. The parameter has a cardinal influence on many physiological processes (e.g. adult mortality, reproduction, respiration, embryonic and gonad development) [20], [4], [21]. Monthly Sea Surface Temperatures (SSTs) data originated from the Hadley centre (http://www.metoffice.gov.uk/hadobs/hadisst/) (Figure 1A).

#### Phytoplankton data

The Continuous Plankton Recorder (CPR) survey has monitored the abundance or occurrence of more than 450 species or taxa in many oceanic and neritic regions of the world [22], [13]. The CPR machine is a plankton sampling instrument designed to be towed by merchant ships or ships of opportunity on their normal sailing routes at a depth of 7 meters and speeds of up to 25 Knots [22]. This machine is operated on a monthly basis since 1948 although the procedure of taxonomic identification was altered in 1958 for zooplankton. The CPR Phytoplankton Colour Index (PCI) constitutes a semi-quantitative representation of the total phytoplankton biomass [22] (Figure 1B). PCI is a rough assessment of the greenness of the CPR silk into 5 categories. The index consistently reflects not only changes in the abundance of phytoplankton on the silk, but also its composition.

#### Zooplankton data

Data on the abundance of *Calanus finmarchicus* and *C. helgolandicus* were provided by the CPR survey (Figure 1C–D). The absence of conventional sampling protocol, the constancy of the sampling depth and the variability of the volume of seawater filtered might have some consequences on our estimations of species abundance. Although zooplankton abundances assessed from the CPR survey are almost always lower than those obtained by vertical net hauls, comparisons made among seasonal and spatial patterns of abundance have always been significant [22]; this has also been the case for *C. finmarchicus* [23]. The constant sampling depth makes the estimations of abundance highly dependent upon sampling hour because of the diel vertical migration of *Calanus* species [24], [25]. The potential effects of diel vertical migration has been ascertained by comparing thermal profiles of *C. finmarchicus* and *C. helgolandicus* using CPR samples collected during daylight (between 10∶00 and 16∶00) and dark periods (between 22∶00 and 04∶00). No significant change in the thermal niche between daylight and dark periods has been identified [12].

## Methods

### Biological Data

All original sets of data used in the study were regularised using as a reference the gridded SST data (99.5°W–19.5°E and 29.5°N–69.5°N; spatial resolution of 1° longitude × 1°latitude). We calculated an arithmetic mean when the number of data for a given cell was >3 CPR samples. We therefore worked on four matrices (120 longitude × 40 latitude × 12 months × 51 years: (1) SST; (2) PCI; (3) *C. finmarchicus*; (4) *C. helgolandicus*.

### Assessment of the 2-dimensional Ecological Niche

**Figure 2:**
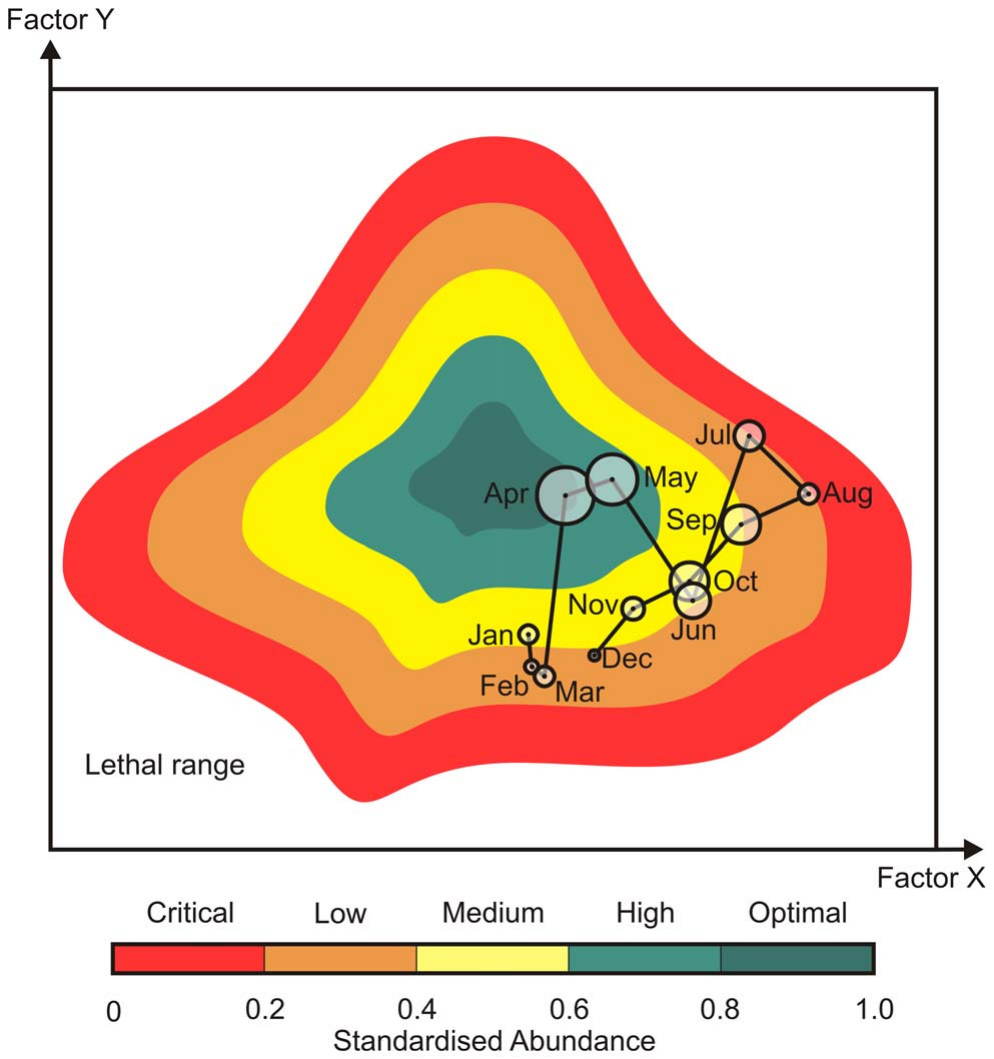
Example of a fictive 2-dimensional realised niche obtained by calculating the standardised abundance of the species (between 0 and 1) for the whole area as a function of both categorised values of environmental factors X and Y. The niche was arbitrarily separated into 5 categories between 0 (lethal range) and 1 (maximum expected abundance). As an example, a set of 12 monthly values of factors X and Y underwent in the North Sea during a fictive year are superimposed in the Euclidean space of the niche (black line). The obtained seasonal trajectory for the species (line) can then be related to changes in its observed abundance (circles).

**Figure 3:**
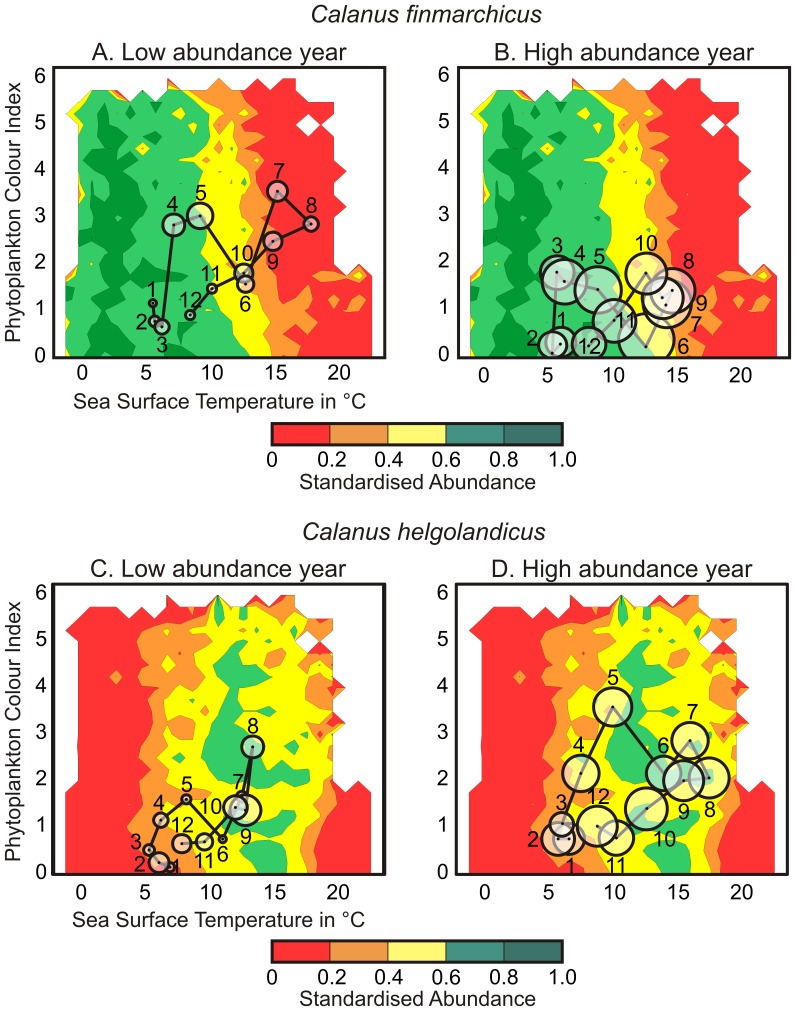
Representation of the environmental conditions (SST and PCI) observed in the North Sea in the niche space for years corresponding to the highest and lowest observed abundance of *Calanus finmarchicus* and *C. helgolandicus*. (A) the warm year 1997 (*C. finmarchicus*); (B) The cold year 1966 (*C. finmarchicus*);. (C) The cold year 1962 (*C. helgolandicus*); (D) the warm year 2003 (*C. helgolandicus*). Monthly abundances of each species were standardised between 0 and 1(see Methods) to allow both seasonal and year-to-year fluctuations to be compared. Seasonal trajectories are represented by lines.

**Figure 4:**
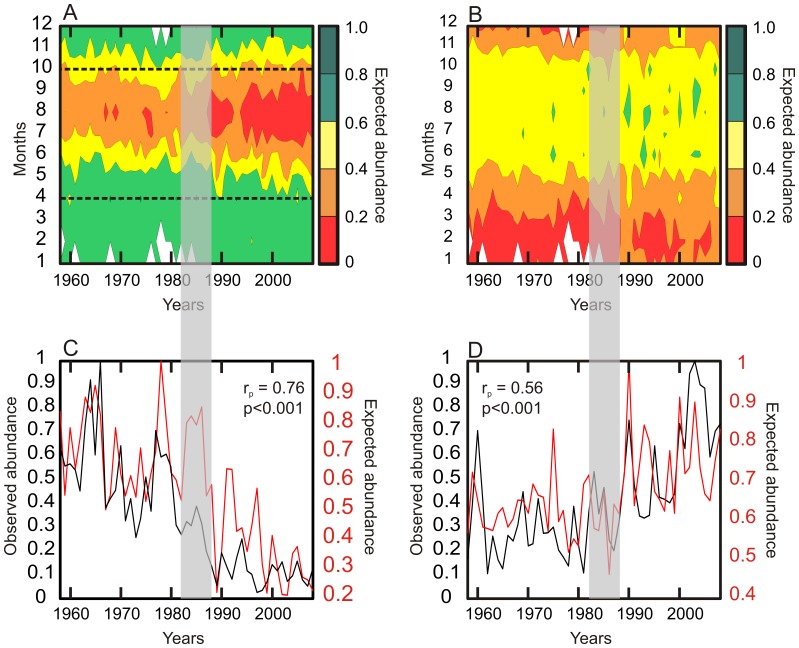
Representation of the long-term changes in the expected abundance of (A) *C. finmarchicus* and (B) *C. helgolandicus* as a function of months and years for the period 1958–2008 (matrix 12×51). (A) Dashed black lines represent the months selected (from April to October) to calculate *C. finmarchicus*‘s expected abundance. For *C. helgolandicus*, all months were included (from January to December). Both observed (in black) and expected (in red) annual mean abundance of (C) *C. finmarchicus* and (D) *C. helgolandicus* are compared using a Pearson correlation coefficient. The period 1982–1988 characterising the abrupt ecosystem shift in the North Sea is represented by a shaded strip.

The ecological niche *sensu* Hutchinson [14] is operationally represented by mapping the abundance of a species as a function of a reduced number of factors, which enable the relative delineation of the species tolerance with respect to its environment. Hutchinson [14] made the distinction between fundamental and realised niche. The realised niche is the fundamental niche once species interactions and dispersal are taken into account. Although the fundamental niche can be determined from the knowledge of the physiology of species [21], the realised niche is more often determined in practice because it can be assessed from the knowledge of the spatial distribution of species, which is the result of the effect of the environment on the physiology and the fitness of the species and includes the influence of species interactions (e.g. competition, predation). The realised niche is commonly assessed in Ecological Niche Models (ENMs) [26]. Figure 2 represents an example using a fictive species. The highest values of abundance, standardised between 0 and 1 (i.e. noted SA as standardised abundance), represent the optimal part of species niche, which allows for successful reproduction. This optimal part was arbitrarily defined by the interval 0.8≤SA≤1. The critical part of the niche 0≤SA<0.2 is associated to low abundances and correspond to extreme conditions that may eventually affect survival [27]. Between these two extremes, the ecological niche 0.2≤SA<0.8 was divided into three intervals: high 0.6≤SA<0.8, medium 0.4≤SA<0.6 and low 0.2≤SA<0.4. When the environment becomes gradually less favourable, it affects consecutively the reproduction, growth and feeding. The same arbitrary intervals and associated colour scales were used in Figures 3 and 4.

Here, we defined the ecological niche of both *C. finmarchicus* and *C. helgolandicus* as a function of monthly Phytoplankton Colour Index (PCI) and monthly SSTs. To avoid any issue of circularity during the next steps of the analysis, the niches were determined using data that originated from the North Atlantic Ocean (2937600 samples), excluding data from the North Sea (107712 samples). Both matrices PCI and SST were divided into categories (26 for SST ranging from −2°C to 25°C every 1°C, 27 for PCI ranging from 0 to 6.5 every 0.25; Figure 3), which were optimised by trial and error, being a compromise between a finer resolution and an increasing number of missing data. The two-dimensional ecological niche was obtained by calculating the averaged abundances for each species and for each category of both parameters. Species abundances were standardised between 0 and 1 by dividing each averaged abundance by the maximum abundance value contained in the matrix 26 PCI×27 SST.

### Superimposing the Environmental Conditions of a given Region on the Niche

Providing that the environmental conditions of a given region are known, we can superimpose the species trajectory in the Euclidean space of the niche (Figure 2, black line). The trajectory of the species can then be related to changes in its abundance (Figure 2, circles). Years corresponding to the minimum and maximum abundances of both *Calanus* were selected (1966 and 1997 for *C. finmarchicus*, Fig. 3A and 3B respectively; 2003 and 1962 for *C. helgolandicus*, Fig. 3C and 3D respectively). For those contrasting years, the monthly averaged values of PCI and SST occurring in the North Sea were superimposed in the niche space to assess the suitability of the area for both *Calanus* (Figure 3).

### Estimation of the Abundance of both Calanus as a Function of PCI and SST for the Period 1958–2008

The abundances representing the two-dimensional ecological niche (North Atlantic Ocean) were standardised between 0 and 1 by dividing the matrix 26 PCI×27 SST by its maximum value. We therefore had a value of standardised abundance corresponding to each category of a lattice defined by two factors. At the scale of the North Sea, this allowed us to attribute a value of expected (standardised) abundance for a given value of both factors for each month of the period 1958–2008. We obtained a matrix containing the long-term monthly changes in the expected abundance in the North Sea (12 months × 51 years) (Figure 4A–B). This procedure enables a monthly quantification of the North Sea suitability for the two *Calanus*.

### Correlations between the Observed and Expected Annual Abundance in the North Sea

The Pearson correlations were calculated between the annual observed and expected abundance of each species (Figure 4C–D). To consider the implications of the diapause of *Calanus finmarchicus* potentially leading to an underestimation of the abundance of the species during the winter months in surface, we recalculated the correlations between the observed and expected abundance using a different number of months. To be consistent, this analysis was also performed on *C. helgolandicus* despite the absence of diapause in its life cycle [16]. The set of months providing the best correlation was selected for each species (Figure 4; Table 1). We therefore expected to obtain the best correlation while the specific set of months selected to calculate the correlations were reflecting the particularity of each species. In other terms, when winter months were removed in *C. finmarchicus* case (i.e. to exclude the diapause period), and when all months were considered for *C. helgolandicus.*

**Table 1:** Pearson correlation coefficients calculated for each species between both standardised observed and expected abundance for the period (1958–2008).

		r (p<0.001)	
First month	Last Month	*C. finmarchicus*	*C. helgolandicus*
January	December	0.55	**0.56**
"	November	0.57	0.52
"	October	0.57	0.5
"	September	0.55	0.48
February	December	0.57	0.54
"	November	0.59	0.49
"	October	0.63	0.47
"	September	0.61	0.45
Mars	December	0.62	0.52
"	November	0.64	0.46
"	October	0.68	0.44
"	September	0.67	0.42
April	December	0.6	0.49
"	November	0.64	0.42
"	October	0.68	0.39
"	September	0.67	0.37
Mai	December	0.65	0.45
"	November	0.69	0.36
"	October	**0.76**	0.33
"	September	0.73	0.31

This was calculated to evaluate the strength of the correlation depending of the period of the year taken into consideration (i.e. between the first month considered and the last one). Maximum correlation values are in bold.

**Figure 5:**
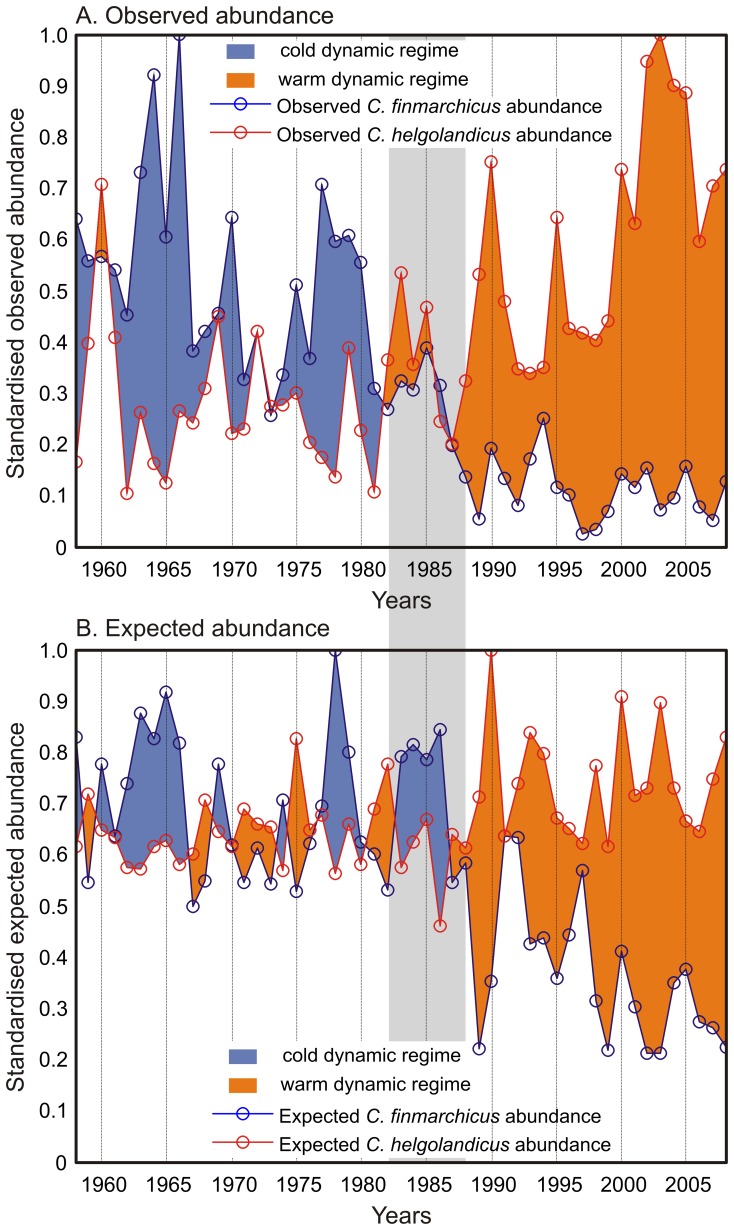
Long-term changes in both the (A) observed and (B) expected abundance of both *C. finmarchicus* and *C. helgolandicus* revealing the different switches from cold- to warm-temperate dynamic regime observed in the North Sea for the period 1958–2008. Positive difference between *C. finmarchicus* and *C. helgolandicus* was represented in blue and a negative difference in red. The period 1982–1988 characterising the abrupt ecosystem shift in the North Sea is represented by a shaded strip.

We then represented the long-term changes in both the observed and expected abundance of both *Calanus* on the same figure. A positive difference between *C. finmarchicus* and *C. helgolandicus* was represented in blue and a negative difference in red to easily identify which species was dominating the North Sea (Figure 5); *C. finmarchicus* being representative of the Atlantic Arctic biome (i.e. cold dynamic regime) while *C. helgolandicus* characterises the Atlantic Westerly Winds biomes (i.e. warm-temperate dynamic regime).

## Results

We assessed the ecological niche of both *Calanus* at the scale of the North Atlantic based on the period 1958–2008. *C. finmarchicus* exhibited high abundances between 0 and 9°C with an optimum range around 3°C (Figure 3A–B). The thermal preferendum of *C. helgolandicus* was higher, ranging from 10°C to 18°C with an optimum value around 11°C. The average thermal regime in the North Sea for the same time period was about 10.4°C, indicating that *C. finmarchicus* was at the edge of its thermal niche while *C. helgolandicus* was close to its optimum (Figure 3). We superimposed the monthly values of both PCI and SST on the 2-dimensional ecological niche for years characterised by the lowest and highest abundances; these years were 1966 and 1997 for *C. finmarchicus* and 2003 and 1962 for *C. helgolandicus,* respectively (Figure 3). Greater values of observed abundance were found for years when the two species were located in the optimal part of their niche. During the year of its lowest abundance (1997), *C. finmarchicus* spent 3 months in the critical part of its ecological niche and its maximum abundance was found in May (Figure 3A). During the year of its highest abundance (1966), *C. finmarchicus* remained above the critical part of its niche and its seasonal maximum was observed in June (Figure 3B). The same patterns were identified for *C. helgolandicus*. When the species spent 6 months within the critical part and the low part of its thermal niche, its overall abundance was low with a seasonal maximum observed in September (1962; Figure 3C). In contrast, its abundance was high when the species remained 9 months above both the critical and low part of its thermal niche, its seasonal maximum being found in October (2003; Figure 3D).

The expected (standardised) abundance of both *Calanus* was then assessed based on PCI and SST at a monthly scale for the period 1958–2008 (Figure 4A–B). The examination of long-term monthly changes in the expected abundance of *C. finmarchicus* showed a decrease of the abundance characterised by an alteration of the environmental conditions placing the species in less optimal part of its niche (Figure 4A). Prior to the North Sea abrupt ecosystem shift, environmental conditions were highly favourable (≥0.6) from November to June while summer environmental conditions corresponded to the low part of the *C. finmarchicus* niche (expected abundance <0.4). After the shift, summer environmental conditions degraded to become more characteristic of the critical part of the niche (<0.2). This alteration was especially pronounced between July and September. The long-term changes in the expected abundance of *C. helgolandicus* were not as pronounced as it was for its congeneric species (Figure 4B). Winter environmental conditions between December and March are decisive for *C. helgolandicus* in the North Sea. After the North Sea abrupt ecosystem shift, these winter conditions improved and the species occurred less frequently in the critical part of its niche. In summer, environmental conditions allow the species to punctually reach the high part of its niche (≥0.6).

We calculated the correlation between the annual observed and the expected abundance based on different combination of months to examine the potential implication of the diapause for *C. finmarchicus* (Table 1) at depths ranging from 500 m to 2500 m [28]. Although all combinations of months gave significant results, the best correlation was achieved when the period from May to October was considered (r = 0.76; p<0.0001; Table 1). The same analysis performed on *C. helgolandicus* revealed that the best correlation was observed when all months were considered (r = 0.56; p<0.0001; Table 1). The graphical examination of the long-term annual changes in both *Calanus* highlighted the strong relationships between the expected and observed abundance (Figure 4C–D). The model overestimates the abundance of *C. finmarchicus* during the abrupt ecosystem shift (1982–1988). The temporary decline in the relationship between expected and observed abundances reduces the overall correlation. This short period in the reduction of the correlation between observed and expected abundance may be related to the increase in the temporal variance of North Sea ecosystems at the time of the abrupt ecosystem shift [5].

We combined the long-term changes in the abundance of the two species to highlight the long-term changes in the observed and expected abundance of both *Calanus* and reveal the modifications of the North Sea ecosystem dynamics (Figure 5). A positive difference between *C. finmarchicus* and *C. helgolandicus* was represented in blue and a negative difference in red. The period prior to the abrupt ecosystem shift was characterised by positive differences in observed abundance associated to the cold-biological dynamic equilibrium (Figure 5A). The differences reduced during the 1980s and became highly negative after the abrupt ecosystem shift onwards. A similar pattern was found with expected abundance, suggesting that environmental conditions were at the origin of the alteration in the observed abundance of the two *Calanus* (Figure 5B).

## Discussion

Here we used a niche-based approach to model the temporal dynamic of *Calanus finmarchicus* and *C. helgolandicus*, thus revealing the role that environmental fluctuations have on their abundances. Despite the use of a limited number of dimensions, this new procedure allowed a simple characterisation of the niche of both species which was precise enough to reflect the degree of mismatch between the environmental conditions and the species’ environmental requirements (i.e. defined as the niche), thereby explaining the long-term changes in the abundance of both copepods in the North Sea. The warming of the North Sea and its pronounced opposite effect on these two key species, appeared mainly in two phases after the 1980s abrupt ecosystem shift and more recently after the mid-1990s. Because the two species are indicative of the position of the boundary between the Atlantic Polar biomes and the Atlantic Westerlies Wind, the decrease in the abundance of *C. finmarchicus* associated to an increase in *C. helgolandicus* reflects an extreme structural reorganisation of the North Sea ecosystem, which is likely to affect both species interactions and ecosystem services [29], [30].

Hutchinson defined the ecological niche as a ‘n-dimensional hypervolume’ in which ‘n’ ideally represents all environmental parameters [14]. Despite that any characterisation of the ecological niche is inherently incomplete, the study showed that it remains possible to accurately model the niche using a reduced number of dimensions providing that those dimensions are correctly chosen (Figure 2). Here, we used two parameters to define the ecological niche of *C. finmarchicus* and *C. helgolandicus*: temperature and an index of food availability (i.e. The Phytoplanktonic Colour Index or PCI). Sea Surface Temperature (SST) has both direct and indirect effects on virtually all aspects of marine ecology (e.g. physiology, biochemistry, ocean physics) and has been frequently used to model species abundance [31], [32], [12]. Furthermore, sea surface temperature covaries with other important variables such as dissolved oxygen, nitrate, phosphate and salinity and consequently integrates several environmental components of the system [15]. Concurrently, food availability essential to the survival of the species has been broadly used in many studies [16], [33]. Ideally, we should use a large number of dimensions to approach with high confidence the ecological niche of a species. However it becomes rapidly unrealistic to use a large number of parameters because only a few can be utilised in an operational way. In contrast to SST and PCI both of which being available on a monthly basis and at a relatively high resolution, dissolved oxygen, salinity and nutrients concentration are not currently measured at these scales and resolutions. Furthermore, Helaouët *et al.* [15] showed that SST and to a lesser extent an index of food quality and quantity was the most important factors that explain the spatial distribution of species. They also found that SST was highly correlated to salinity, nutrients concentration and dissolved oxygen. Therefore, SST also represents a proxy for these parameters. The less parameters used, the less the influence of both error measurements and estimations on the calculated expected abundance.

We standardised the species abundance as a function of the two environmental parameters to obtain a representation of the ecological niche. The balance between the resolution of the niche and the quality of its estimation (including the number of missing values) has a direct influence on the expected abundance we calculate. A low-resolution niche has larger categories of both SST and PCI. The resulting cells contain more samples, which increase the quality of the estimation. However, the contour of the niche becomes less precise. Conversely, when the resolution becomes too high, the number of cells where the estimation is not possible because of lack of sampling also increases. From the knowledge of the environmental conditions, we then calculated the expected (standardised) abundance and compared it to changes in the observed (standardised) abundance of *Calanus*. Both observed and expected standardised abundances were subsequently divided into 5 categories: critical, low, medium, high and optimal.

The procedure offered a simple way to quantify, for each month, the degree of suitability of the North Sea toward the requirement of both *Calanus* species. Using an approach based on the concept of the ecological niche of Hutchinson, our analyses also revealed that the long-term changes in the abundance of the two *Calanus* are linked to an alteration of the North Sea environmental conditions related mainly to temperature. The study highlighted that in the North Sea the unfavourable period for *C. finmarchicus* is summer whereas it is winter for *C. helgolandicus*. The North Sea warmed by 1°C between the 1960s and the 2000s [34], which has established a new period when conditions have become critical for *C. finmarchicus.* These results explained the long-term diminution observed by several authors in the North Sea [35], [13]. Many hypotheses have been proposed to explain the long-term fluctuations in the abundance of this species. Fromentin & Planque [36] found that the abundance of *C. finmarchicus* was negatively correlated to the state of the North Atlantic Oscillation (NAO). They hypothesized that the link between this species and this atmospheric oscillation occurred via NAO-induced modifications in the sea productivity, which then modified the interspecific competition between *C. finmarchicus* and *C. helgolandicus*. Although subsequently Planque & Reid [37] argued that the relationship broke-down in 1996, new findings have revealed the relationship stopped at the time of abrupt ecosystem shift in the North Sea [38], [39], [40]. According to our findings, these authors proposed that temperature was the main driver of the decline in *C. finmarchicus* and the increase in *C. helgolandicus*. Another hypothesis is based on the spring invasion of the species in the northern part of the North Sea, which depends on the volume of the Norwegian Sea Deep Water and the amount of the Atlantic inflow into the North Sea [13]. These studies have provided evidence that the magnitude of the spring invasion has been reduced due to the warming of the Norwegian Sea Deep Water [41]. Helaouët & Beaugrand [12] provided evidence that the two species of *Calanus* were extremely sensitive to the thermal regime of the North Sea. Using the ratio *C. helgolandicus* on both *Calanus* as a function of months and sea surface temperature, they explained the strong decline of the subarctic copepod in the North Sea by solely using changes in monthly temperatures from 1960 to 2002.

Many biological changes have been observed after the abrupt ecosystem shift in the North Sea during the 1980s [42], [43]. Our results showed that both *Calanus* exhibited an abrupt change *circa* 1987 (Figure 4, 5). The study provides evidence that the decline in *C. finmarchicus* after the shift is related to the appearance of a critical period in summer (see Figure 4A). After ∼1987, the species spent a significant amount of time in summer in the critical part of its ecological niche (see Figure 2, 3), which might not only affect the reproduction [44], but also the survival [21]. Although the species might react by moving into deeper waters to alleviate the effects of rising temperature [45], this effect has not been yet demonstrated in field at the scale of the North Sea. In contrast, this increase in sea temperature has influenced positively the abundance of *C. helgolandicus*. The effect of temperature has been to fragment the critical period observed in winter prior the abrupt ecosystem shift (see Figure 4B). This has probably enabled an increase in winter survival, which might explain why the species is identified in spring after the shift [13], [16]. In summer, environmental conditions improved after the shift bringing occasionally the species closer to the optimal part of its niche (Figure 4B). Increased temperatures in winter and summer probably explain the strong positive deviation in the abundance of *C. helgolandicus* observed in the North Sea [46].

Because both *Calanus* are an important component of the North Sea ecosystem, our study suggests that the dynamic regime has been substantially altered by the warming of this area (see Figure 5). These major modifications paralleled a northward movement of a critical thermal boundary of 9–10°C, which is a proxy of the transitional zone separating the Atlantic Polar and the Atlantic Westerly Wind biomes [9], [5]. This biogeographical movement affected many species important for the ecology of the North Sea from phytoplankton to zooplankton and fish [34], [43], [47]. Another abrupt change might have occurred after the mid-1990s as a consequence of a substantial increase in temperature [48], [49]. This shift has been detected in the southern part of the North Sea and in the Bay of Biscay on plankton, fish and might have also influenced the northwards movement of the Balearic shearwater *Puffinus mauretanicus* [50]. Although our procedure explains well changes in the abundance of *Calanus*, observed patterns of abundance are more amplified than those assessed by our procedure. This discrepancy is probably the results of species interaction, which is likely to precipitate the decline of the species, a phenomenon termed trophic amplification [47].

Currently one of the major problems in understanding long-term changes in populations or community is deciphering the many environmental changes that impact on them, be it regional climate variability or a combination of many anthropogenic factors. Here we have used a niche approach to quantify the role that climate variability has directly on plankton populations. While the focus of this study was on two marine zooplanktonic species, the method can be extended to other species from plankton to fish and seabirds. Similar approaches including Ecological Niche Models, also known as Species Distribution Models, have been applied on many different types of species [51], [26]. However as with other methods, our procedure necessitates to clearly identify the main parameters controlling the distribution of the species. When species are exploited (e.g. fish) or affected by other human activities (e.g. sea turtles and mammals), this becomes more complicated because the spatial and temporal patterns exhibited by the species may in large part be related to human activities, which can significantly affect expected abundances. In the case of fish, the procedure can nevertheless be used in an attempt to decipher changes related to environmental changes from those related to overharvesting [52].
